# Markers of Oxidative Stress and Neuroprogression in Depression Disorder

**DOI:** 10.1155/2015/898393

**Published:** 2015-05-20

**Authors:** Magdaléna Vaváková, Zdeňka Ďuračková, Jana Trebatická

**Affiliations:** ^1^Institute of Medical Chemistry, Biochemistry and Clinical Biochemistry, Faculty of Medicine, Comenius University, Sasinkova 2, 813 72 Bratislava, Slovakia; ^2^Department of Pediatric Psychiatry, Medical Faculty, Comenius University and Child University Hospital, Faculty of Medicine, University Hospital, Limbová 1, 833 40 Bratislava, Slovakia

## Abstract

Major depression is multifactorial disorder with high prevalence and alarming prognostic in the nearest 15 years. Several mechanisms of depression are known. Neurotransmitters imbalance and imbalance between neuroprogressive and neuroprotective factors are observed in major depression. Depression is accompanied by inflammatory responses of the organism and consequent elevation of proinflammatory cytokines and increased lipid peroxidation are described in literature. Neuropsychiatric disorders including major depression are also associated with telomerase shortening, oxidative changes in nucleotides, and polymorphisms in several genes connected to metabolism of reactive oxygen species. Mitochondrion dysfunction is directly associated with increasing levels of oxidative stress. Oxidative stress plays significant role in pathophysiology of major depression via actions of free radicals, nonradical molecules, and reactive oxygen and nitrogen species. Products of oxidative stress represent important parameters for measuring and predicting of depression status as well as for determining effectiveness of administrated antidepressants. Positive effect of micronutrients, vitamins, and antioxidants in depression treatment is also reviewed.

## 1. Introduction

Major depression is a mental disorder that occupies fourth position of worldwide disability list and is expected to become the second most frequent disease by 2030 [1]. Major depression not only decreases productivity and quality of life of patients but also represents a significant financial burden for health care [2, 3]. Depression is multifactorial disorder and its etiology includes genetics, environmental, psychological, and biological factors. Several molecular mechanisms play role in pathogenesis of depression. Depression is caused by abnormalities in metabolism of neurotransmitters (e.g., serotonin and dopamine) that is consequently affected by enzymes involved in their degradation (e.g., monoamine oxidase) or synthesis of their precursor tryptophan. To increase concentration of serotonin in the synaptic cleft is successful strategy for treatment of major depression via selective serotonin reuptake inhibitors (SSRI) antidepressants. SSRI represents the most frequently used antidepressants for treatment of major depression (88.5%) [2, 4–6]. Major depression is also associated with inflammatory processes and elevated levels of proinflammatory cytokines, decreased neurogenesis, and subsequent neuroprogression (pathological reorganization of the central nervous system), mitochondrial dysfunction, and dysfunction of hypothalamic-pituitary-adrenal axis. There are observed lowered antioxidant concentrations and increased oxidative stress levels as well [5, 7–9].

Oxidative stress is connected with many diseases including atherosclerosis, cardiovascular and neurodegenerative diseases, like Alzheimer's disease and Parkinson's disease, psychiatric disorders like Attention Deficit Hyperactivity disease and schizophrenia, or cancer, diabetes mellitus, and others. Oxidative stress is caused by imbalance between prooxidant and antioxidant in favor of prooxidant. Oxidative damage to cells and organs can be caused by free radicals (FR) (superoxide, hydroxyl radical) or nonradical molecules, like hydrogen peroxide, and their derivatives called reactive oxygen species (ROS) and reactive nitrogen species (RNS). They are expressed in the organism during standard metabolism. Mild oxidative stress and free radicals play an important role in the regulation of many processes in the organism, for example, during phagocytosis, apoptosis, egg fertilization, or activation of certain transcriptional factors or in cell signaling pathways [10–13].

However, if ROS and RNS are produced in large amount and in the wrong place, they can cause oxidative modifications of lipids, proteins, and DNA [9, 14–16]. They can modify cell membranes and function of receptors and alter enzymes and genes activity. Oxidative stress contributes also to the aging [8, 11, 16–20]. Direct measurement of ROS concentration is difficult because of its extremely short half-lives. Obvious alternative option is to measure products of oxidative stress in proteins, lipids, or nucleic acids [12, 21].

To fight excessive production of ROS and RNS, the organism has built protective systems and mechanisms against their toxic effects. Protection is organized at three levels: (a) systems preventing FR formation, such as inhibitors of enzymes catalyzing FR formation. (b) When these primary protective systems are insufficient and FR and ROS are already formed, scavengers and trappers of FR come into action and eliminate high reactivity of ROS by turning them into nonradical and nontoxic metabolites. These compounds are called antioxidants and they prevent oxidation of biologically important molecules by FR or ROS. (c) If protection of the organism fails at this level, then repair systems recognize impaired molecules and decompose them, as it is in case of proteinases at oxidatively modified proteins, lipases at oxidatively damaged lipids, or DNA repair systems at modified DNA bases [13].

## 2. Neuroprogressive Processes and Hormones Activity in Depression

**Figure 1:**
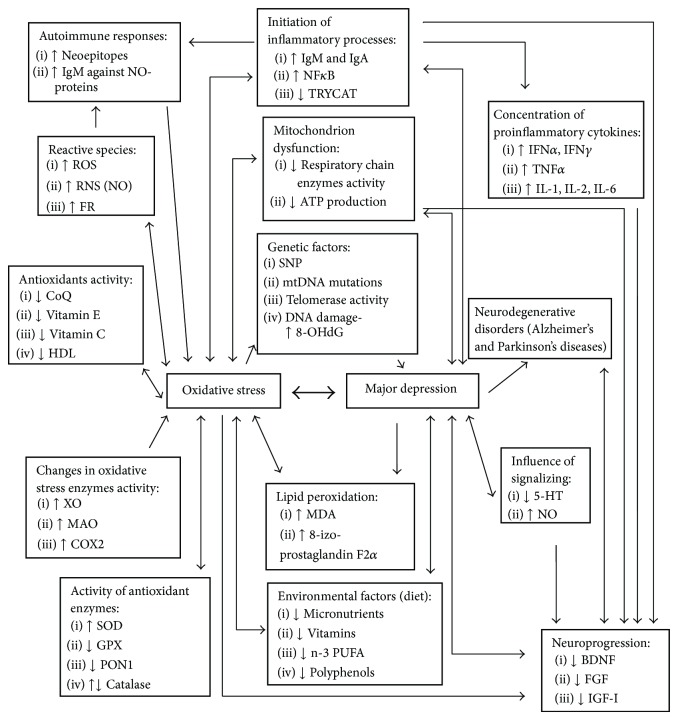
Schematic relations between oxidative stress and depression formed in patients with depression in comparison to healthy controls. Abbreviations: 5-hydroxytryptamine (5-HT), 8-oxoguanine (8-oxoG), 8-oxodeoxyguanosine (8-oxodG), 8-iso-prostaglandin F2 alpha (8-iso-P), brain-derived neurotrophic factor (BDNF), coenzyme Q10 (CoQ10), cyclooxygenase 2 (COX2), fibroblast growth factor (FGF), free radicals (FR), glutathione peroxidase (GPX), high density lipoprotein (HDL), interferon (IFN), insulin-like growth factor 1 (IGF-I), immunoglobulin (Ig), interleukin (IL), monoamine oxidase (MAO), malondialdehyde (MDA), mitochondrial DNA (mtDNA), omega 3 polyunsaturated fatty acids (n-3 PUFA), nuclear factor *κ* B (NF*κ*B), paraoxonase 1 (PON1), reactive nitrogen species (RNS), reactive oxygen species (ROS), single nucleotide polymorphism (SNP), superoxide dismutase (SOD), tumor necrosis factor alpha (TNF*α*), tryptophan catabolites (TRYCAT), xanthine oxidase (XO).

Balance between neuroprogressive and neuroprotective factors is established by grown and neurotrophic factors. Their expressions are also affected by stress (Table 1, Figure 1) [5].

Brain-derived neurotrophic factor (BDNF) is the most abundant neurotrophic factor in brain. Lowered concentration in serum is typical state for patient with depression and bipolar disorder. There is also correlation between changes in concentration of BDNF and severity of illness. Lowered concentration of BDNF increased level of oxidative stress which consequently affects expression, folding, and secretion of BDNF. These changes lowered neurogenesis of brain cells. An antidepressants usage increases concentration of BDNF and stimulates neurogenesis and regeneration of cells [5, 7, 22–25]. On the other hand, some studies fail to detect changes in concentration of BDNF in plasma between depressed patients and control group and between patients on antidepressants and medicine-free patients [68].

Probability of development of depression is increased with a lower level of insulin-like growth factor (IFG-I) in brain. An exact mechanism of how IFG-I influences depression is unknown. It is assumed that metabolic pathway of lithium efficacy in treatment of bipolar disorder is involved [69, 70].

Nuclear factor *κ* B (NF*κ*B) is transcription factor that is also connected with development of depression. Proinflammatory cytokines (e.g., interleukin 6 and interleukin 8) are activated by NF*κ*B. Activity of this transcription factor is affected by levels of ROS and glutamate. NF*κ*B itself increases oxidative stress in the organism and causes inflammatory reaction which can lead to neuroprogression [5, 8, 70]. Increased levels of NF*κ*B were observed in depressed patients [38].

An imbalance between production and release of* neurotransmitters*, like dopamine, serotonin (5-hydroxytryptamine), glutamate, or noradrenalin in brain, is also associated with depression and neurodegenerative disease [4, 71].

Brain levels of neurotrophic factor 5-hydroxytryptamine (5-HT) affect second messenger signalization, expression of BDNF, and receptors for serotonin transporters. Lowered sensitivity of 5-HT receptors was detected in depressed patients and it is caused by inhibition of neurogenesis of 5-HT neurons [5, 26].

Secretion of melatonin affects a circadian cycle. Several depression symptoms, like restless sleep, early morning awakening, sleeplessness, daytime fatigue, and moodiness, are caused by interfering of day and night cycle. An antidepressant effect was recorded only after administration of agomelatine, a melatonin analogue. Improvement of patients with depression was markedly only in severe cases of depression. An antidepressant effect of agomelatine is presumably caused by synergic effect of melatonin (receptors MT_1_, MT_2_) and monoamine (receptors 5-HT_2C_) [4, 72–75].

Tryptophan catabolites (TRYCATs) are created by tryptophan breakdown by enzyme indoleamine 2,3-dioxygenase (IDO). Tryptophan, as a precursor of serotonin, is an important marker of depression. Its lowered level can lead to inflammatory reaction of the organism. IDO activity is negatively correlated with concentration of serotonin and tryptophan and is positively correlated with depression severity. Lowered levels of TRYCATs and tryptophan are also associated with melancholic depression. TRYCATs, like kynurenine, kynurenine acid, or xanthurenic acid, possess neuroprotective but also depressogenic, anxiogenic, and neurotoxic properties [4, 5, 61–64, 76].

Nitric oxide (NO), if it is produced in access, is strong damaging-free radical. However, at low concentration NO is considered to be an important neurotransmitter connected to pathophysiology of depression, anxiety, epilepsy, and schizophrenia. NO is synthesized from L-arginine through constitutive and endothelial NO synthase (cNOS, eNOS) at physiological conditions. At some pathological conditions an inducible NO synthase (iNOS) forms high concentration of NO. However, at low concentration, NO acts as second messenger in the process of dopamine and noradrenalin releasing. NO affects sexual and aggressive behavior and via synthesis pathway also takes part in anxious behavior, inflammation, and depression elicited by interferon alpha (IFN*α*) [8, 17, 74, 77, 78]. Even temporary elevated levels of NO cause nitration and hypernitrosylation of amino acids and proteins. That leads to creation of highly reactive substances, for example, NO-tyrosine, NO-tryptophan, NO-arginine [79, 80], and NO-bovine serum albumin (NO-BSA) [81]. Indeed, Maes et al. [82] detected neoepitopes and anchorage epitopes created by autoimmune response of IgM against palmitic and myristic acids and also elevated levels of IgM against NO-tyrosine, NO-aspartate, and NO-phenylalanine in serum of depressed patients [82]. Elevated concentration of NO was detected in patients with depression and in patients with suicidal thoughts compared to nonsuicidal patients [27, 28]. Antidepressant-like effect can be induced by inhibition of NO synthesis in brain. Inhibition of NOS can lead to increased effectiveness of serotonergic antidepressants and can be applied to patients suffering from drug resistant depression [8, 17]. Eight weeks of antidepressants administration leads to lowered concentration of NO [33].

## 3. Effect of DNA Damage on Depression

An excessive telomerase shortening is observed in several neuropsychiatric disorders like depression, schizophrenia, anxiety, and affective disorder, in consequence of lowered activity of telomerase. Length of telomeres is negatively correlated with longitude of nonmedicated depression and concentration of interleukin 6 (IL-6). IL-6 is considered to be a marker of inflammation. An exact mechanism of telomerase shortening and premature aging of the organism is unknown. Hypothesis said that oxidative stress and inflammation affect structure of replication fork in the vicinity of telomeres. Possibility is also that oxidative stress induced DNA damage preferably in telomeres in comparison to nontelomeric DNA [7, 8, 29, 30].

Oxidative changes in nucleotides are caused by ROS and RNS. 8-Oxodeoxyguanine (8-OxoG) is formed by oxidation of guanine and is diluted by urine. In urine of depressed patients significantly elevated levels of 8-OxoG are detected in comparison to healthy control [12, 17, 31, 32]. Another predisposition of depression can be a specific single nucleotide polymorphism (SNP). Change of nucleotide sequence can decrease activity of peptidases or influence several genes connected to metabolism of ROS and RNS. SNPs in genes for IL-1, IL-6, tumor necrosis factor alpha (TNF*α*), cyclooxygenase 2 (COX2), superoxide dismutase (SOD), and catalase is associated with depression [17, 61, 83]. SNPs in serotonergic genes can also alter antidepressants responses [84].

## 4. Oxidative Stress-Related Enzymes in Depression

Changes in activity of oxidative stress-related enzymes can be caused by depression and inflammation (Table 1, Figure 1). Several enzymes involved in ROS production showed elevated levels in depressed patients in comparison to nondepressed controls [7, 17].

Xanthine oxidase (XO) represents enzyme that catalyzes oxidation of xanthine. Products of this reaction are superoxide and hydrogen peroxide. Increased serum level of XO was observed in depressed patients and in thalamus brain area in postmortem depressed patients [5, 17, 33–36]. Oxidative deamination of monoamine neurotransmitters and metabolism of serotonin is affected by monoamine oxidase (MAO). Its byproducts (e.g., hydrogen peroxide) cause overproduction of ROS that can lead to neuron apoptosis and mitochondrial dysfunction [4, 7]. Elevated level of MAO was also detected in patients with depression and* postpartum depression* [37]. COX-2 represents another enzyme that takes part in inflammatory processes in the organism.* COX-2 inhibitors cause neuron inflammation* that can consequently lead to worsening depression and increase probability of cardiovascular diseases. They can also damage mitochondrion, increase lipid peroxidation, and decrease antioxidant concentration [8, 9, 85]. Enzyme NADPH oxidase is associated mainly with production of superoxide by neutrophils during phagocytosis. In neurons NADPH oxidase positively correlate with processes of inflammation too. Inflammation-induced activation of microglial NADPH oxidase and iNOS has been reported to act synergistically to kill neurons through the formation of peroxynitrite. Peroxynitrite is a potent oxidant with biological reactivity similar to that of the hydroxyl radical. Expression of both enzymes is regulated by transcriptional factor NF*κ*B [9, 86].

Activity of antioxidant enzymes was found controversial in patients with depression. In some studies increased activities were detected but, on the other hand, several studies published mixed or negative results of enzymes activity in depression in comparison to healthy control groups (Table 1, Figure 1) [38, 39, 41]. The contradiction can be caused by small study sample, heterogeneity of patients' statuses, or variability in individual experiments [8].

Superoxide dismutase (SOD) with its cofactors (zinc and copper) catalyses break down from superoxide to oxygen and paradoxically to hydrogen peroxide which is at physiological conditions decomposed by catalase. An elevated level of SOD is observed as organism response to elevated concentrations of superoxide [7, 17]. In depressed patients increased and also decreased activity of SOD was shown [38–40]. Glutathione peroxidase (GPX) catalyzes reduction of peroxides, including hydrogen peroxide. GPX represents one of the main antioxidant enzymes in the organism [87]. In patients with affective disorder, depression and schizophrenia detected lowered activity of GPX in comparison to healthy control. Decreased activity causes accumulation of ROS and negatively correlates with severity of depression and its autonomous symptoms. GPX also protects the organism against cell death, DNA, and neurons damage [17, 40–42, 76]. Some studies on the other hand did not find significantly different level of GPX in depressed patients [38, 39]. Digestion of hydrogen peroxide to water and oxygen is catalyzed by enzyme catalase. Cell proliferation, apoptotic signals, and thrombocytes activation are influenced by catalase activity. Elevated activity of catalase was observed in patients in acute phase of depression and also in patients with bipolar disorder on lithium medication [39, 88, 89]. On the other hand, other works detected lowered activity of catalase in depression [43]. Oxidative stress and inflammation also affect activity of paraoxonase 1 (PON1). PON1 is responsible for antioxidant properties of HDL and protects lipids against oxidative damage through lipoperoxidation. Together with HDL it possesses antiatherogenic properties [90]. Activity of PON1 is lowered in major depression and its activity also negatively correlates with smoking [44]. Other work did not find changes in PON1 activity in depressed women [40].

## 5. Effect of Antioxidants, Micronutrients, and Vitamins in Depression

Compounds capable of prevention or suppression of reaction between substrate and FR or ROS and RNS are defined as antioxidants. Antioxidants are able to be effective at very low concentrations and by ratio of 1 : 100 to amount of free radicals. Antioxidants in the organism are endogenous (e.g., glutathione, uric acid) and exogenous, like vitamin C, vitamin E, and polyphenols. An abnormal prefrontal level of GSH, the major cellular redox regulator and antioxidant in peripheral tissues and brains of patients with schizophrenia and bipolar disorder, was observed. Lowered concentration of antioxidants in the organism causes elevated production of ROS that leads to fatty acids membranes disruption and also to damage of proteins and DNA. Their concentration can be affected by diet (Table 1, Figure 1). Appraisal of antioxidant activity of natural substances is hindered by disagreement between results observed* in vivo* and* in vitro* [4, 7, 13, 61, 91].

Polyphenols are products of secondary metabolism of plants. They possess ability to absorb free radicals and to bond prooxidant ions of metals. They can increase activity of antioxidant enzyme or also increase expression of BDNF. There are observed anti-inflammatory effects of polyphenols. Increased cognitive abilities, mood improvement, and lowered probability of neurodegenerative disorders are accredited to diet rich in polyphenols. Among polyphenols are, for example, kurkumine, EGCG (epigallokatechin-3-gallat), resveratrol (presented in red wine, grapes, berries, and peanuts), chlorogenic acid (detected in apples, plums, and cherries), or EGb761 (detected in* Ginkgo biloba*) [6, 7, 13, 92, 93]. Kurkumine is contained in roots of* Curcuma longa*. It prevents lipid peroxidation and scavenges ROS and RNS. Apart from anti-inflammatory and antioxidant effect it is described also as having antiviral, antifungal, and antiamyloidogenic properties. It inhibits concentration decrease of 5-HT_1A_ and BDNF, normalizes concentration of serotonin and dopamine, and inhibits activity of COX-1, COX-2, MAO-A, and MAO-B genes [7, 92, 94]. EGCG represents a main component of green tea (>20%). It possesses the ability of decreasing oxidative stress and TNF*α* production. Frequent green tea drinking can lower probability of depression occurrence and has potential to decrease symptoms of depression and stress and also increases cognitive abilities in consumers [7, 92]. Antidepressant-like effect was also confirmed in substance EGb761 found in* Ginkgo biloba* by affecting serotonin and dopamine concentrations [7]. Combined therapy with antidepressants and EGb extract increases sleep efficacy and reduces awakenings [95]. Polyphenolic extract from pine bark* Pinus pinaster*, Pycnogenol, improves symptoms of ADHD, normalizes level of neurotransmitters, and decreases oxidative stress markers [96–99].

Omega 3 polyunsaturated fatty acids (n-3 PUFA) can be found in sea food, fishes, and olive oil in high concentrations. Increased probability of depression and postpartum depression is associated with lowered food supply of n-3 PUFAs. In patients with depression, chronic fatigue and bipolar disorder are observed to have significantly decreased level of n-3 PUFA in comparison to healthy controls [61, 100–102]. Combined therapy of SSRI antidepressants with n-3 PUFA indicated to be more effective than antidepressants monotherapy [5, 103]. Consumption of at least one fish a week also decreases cognitive capability decline in old age approximately about 10–13% [104]. Other research, on the other hand, detected only small and nonsignificant effect of n-3 PUFA in depression [105, 106]. A number of accomplished suicides are also not significantly affected by n-3 PUFA consumption [107]. Among n-3 PUFAs are mainly eicosapentaenoic acid (EPA) and docosahexaenoic acid (DHA). DHA represents the main compound of phospholipid membrane in the brain. It inhibits apoptosis of neurons and ensures transfer of serotonin, norepinephrine, and dopamine. DHA also possesses antioxidant and antidepressant properties. EPA influences efficacy of immune system, decreases concentration of arachidonic acid (AA) in cell membranes, and lowers synthesis of prostaglandin E and cytokines like IL-1, IL-6, TNF*α*, and IFN*γ*. Consumption of EPA has significant antidepressive and anti-inflammatory effects. Omega 6 (n-6) PUFA, like AA, creates ROS by increasing proinflammatory signal molecules levels, for example, eikosanoids. n-6 PUFA promotes proinflammatory effects in the organism and increases production of IL-1, IL-6, and TNF*α*. It also directly induces brain tissue apoptosis [8, 18, 19, 108].

Coenzyme Q10 (CoQ10) represents an important antioxidant with anti-inflammatory and neuroprotective properties. It inhibits proinflammatory cytokines production and suppresses expression of NF*κ*B. Lowered level of CoQ10 is associated with increased concentration of TNF*α*, ROS, and RNS and also mitochondrion dysfunction. Patients with drug resistant depression show lowered concentration of CoQ10 in comparison to healthy controls [5, 17, 45, 109, 110].

N-Acetyl cysteine (NAC) is an antioxidant that effectively reduces hydroxyl radicals. Intake of NAC increases concentration of cysteine in plasma and affects glutathione (GSH) levels. It also affects levels of glutamate and dopamine releasing. NAC has shown to possess anti-inflammatory properties and decreases levels of IL-1*β*, IL-6, and TNF*α* [7, 8]. Administration of NAC to patients with depression as part of bipolar disorder causes significant decrease of depression severity and improves antidepressants effectiveness [111, 112].

High density lipoprotein cholesterol (HDL) affects inflammatory and immune reaction of organism [113]. Lower level of HDL is associated with higher atherogenicity and risk for cardiovascular disease [90, 114]. Atherogenesis is frequently accompanied by depression in women [49]. Lowered serum level of HDL is detected also in depression, especially in patients with suicidal attempts [113] and with postpartum depression. There is also negative correlation between HDL concentration in serum and phobia, anxiety, and depression in women [115] and severity of postpartum depression [116]. On the other hand, no association between depression and level of HDL was found in elderly men [117]. Antidepressant treatment does not influence concentration of HDL in patients [113].

Depression is associated with deficiencies of some elements. For example, deficiency of magnesium causes mild inflammation. Its shortage also increases production of ROS and NO that can cause symptoms of depression especially in elderly people. In addition, intake of magnesium was associated with antidepressant-like effect in patients with depression [74, 118]. Besides magnesium deficiency, depression is usually accompanied also by iron shortage [41, 119]. Selenium is a main component of selenoproteins and amino acids, like selenomethionine and selenocysteine. Several selenoproteins, for example, GPX and thioredoxin reductase, possess antioxidant properties. They decrease oxidative damage of DNA and lower oxidative stress, increase expression of tumor suppressor p53, inactivate protein kinase C, and inhibit angiogenesis. Lowered level of selenium (62 *μ*g/L) can be responsible for depressive mood, anxiety, and decline of cognitive function. Dietary intake of selenium can lower probability of postpartum depression and decrease depression severity. On the other hand, very high selenium status (110 *μ*g/L) is also associated with depression [7, 50–53]. Deficiency of zinc is also associated with depression. It causes cognitive decay and humoral and cell immunity malfunction. Level of zinc also affects activity of antioxidant enzyme (Cu/Zn SOD) and enzymatic pathways of metalloenzymes dependent on zinc. Its concentration is important for homeostasis and signal transduction. It stimulates neurogenesis by increasing BDNF expression and by inhibition of glycogen synthase kinase 3 prevents neuroprogression. Deficiency of zinc increases oxidative stress and also concentrations of inflammatory cytokines. Zinc administration is efficient not only as prevention against symptoms of depression but also as effective antidepressant in postpartum depression and drug resistant depression. When combined with antidepressants usage, it increases efficacy of medication [5, 17, 54, 55, 120].

Deficiency of several nonenzymatic antioxidants is connected with worsening depression severity and anxiety. For example, significantly decreased level of vitamin E (*α*-tokoferol) was found in depressed patients in comparison to healthy controls [46]. Its concentration is negatively correlated with depression severity and also with levels of leukocytes and neutrophils. Antioxidant properties of vitamin E protect organism against lipid peroxidation. Diet rich in *α*-tokoferol also increases concentration of GSH and activity of glutathione reductase [7, 17, 121, 122]. In patients with depression lowered concentration of ascorbic acid (vitamin C) was also observed. Its intravenous administration not only amplifies antidepressants efficacy, but also act as antidepressant itself [7, 46–48]. Depression is associated with shortage of folate and vitamin B12 levels. Their deficiency affects metabolism of monoamines that can worsen depression severity in patients [123–126].

## 6. Effect of Mitochondrial Dysfunction on Depression

Production of ATP in mitochondrion insures most of the energetic demand of the organism. This process is associated with excessive production of ROS and increasing of oxidative stress (Figure 1). Disrupted function of mitochondrion and an insufficient oxygen radical degradation increase concentration of ROS in the organism that consequently causes lipid, protein, and DNA damage. Depression is associated with decreased activity of enzymes involved in respiratory chain, decreased ATP production, and other mitochondrial dysfunctions caused by deletion in mitochondrial genome [5, 8, 61, 127, 128].

## 7. Correlation of Depression and Inflammation

Increased concentration of proinflammatory cytokines TNF*α*, IFN, and IL can lead to oxidative damage. Overproduction of proinflammatory cytokines can be related to insufficient activity of antioxidant enzymes and low level of antioxidants. Overproduction can lead to pathological changes in brain that can escalate to cognitive dysfunction or neuropsychiatric disorders like schizophrenia, anxiety, depression, and depression states (lethargy, sleep disorders, weight loss, or anorexia). Levels of cytokines influence also neuroendocrine function and neurotransmitter metabolism. Imbalance of proinflammatory and anti-inflammatory cytokines can be a trigger of neurodegenerative disorders, for example, Alzheimer's or Parkinson's diseases. Inflammation processes in the organism are negatively correlated with concentration of antioxidants and antioxidant enzymes (Table 1, Figure 1) [5, 61, 129, 130]. In patients with depression and anxiety inflammatory reaction and mild chronic inflammation were observed to be caused by increased concentration of proinflammatory cytokines like TNF*α*, IL-1*α*, IL-1*β*, IL-4, IL-5, IL-6, IL-12, IFN*γ*, and C-reactive protein. Increased levels of inflammatory positive acute phase proteins, for example, haptoglobin, *α*1-antitrypsin, and ceruloplasmin, a *α*1-acid glycoprotein, were also detected. Antidepressants themselves possess anti-inflammatory effect by, for example, decreasing production of IFN*γ* and IL-12. In case of neurodegenerative disorders that are associated with chronic inflammation antidepressants act as preventives. In addition, inhibition of proinflammatory cytokines or their signal pathways increases effectiveness of antidepressants and decreases depression state in patients [4, 5, 7, 17, 41, 56–61, 127].

Depression is often accompanied by inflammation of digestive tract and increased number of gram-negative bacteria and Enterobacteria (e.g.,* Pseudomonas aeruginosa*,* Morganella morganii*,* Pseudomonas putida*, and* Citrobacter koseri*). Their presence causes activation of immune response against lipopolysacharides (LPS) of gram-negative bacteria especially by increasing immunoglobuline (Ig) A and IgM concentrations [5, 131, 132]. Immune reaction mediated by IgM towards phosphatidylinositol [133], NO-BSA [81], and NO-cysteinyl [134] is detected in serum of patients with chronic fatigue syndrome. IgM responses against oleic acid and phospatidylinositol are also negatively correlated with severity of symptoms [134]. There also is recorded higher IgM response against NO modified amino acids, for example, NO-tyrosine, NO-arginine, and NO-tryptophan in serum of patients with chronic fatigue syndrome in comparison to healthy volunteers [79]. Major depression is also accompanied by elevated serum levels of IgM and autoimmune response against NO-tyrosine and oleic acid especially in chronically depressed patients. Antidepressants usage has no significant effect on IgM response [65].

## 8. Lipid Peroxidation in Depression

Increased lipid peroxidation is associated with depression. Lipid peroxidation is caused by increased concentration of proinflammatory cytokines that produce free radicals and increase metabolism of monoamines. Lipid peroxidation decreases life span of neurons, lowers expression of neurofilaments, reduces stability of membranes and activity of ion channels, affects neurotransmitter release, and by malondialdehyde (MDA) affects connection of 5-HT and membranes (Table 1, Figure 1). It belongs among important markers of cell damage caused by oxidative stress [7, 17, 135]. Decreased lipid peroxidation can be achieved by antidepressants and antioxidants (e.g., vitamin E) administration [39, 136].

Malondialdehyde (MDA) represents an end product of PUFA and AA peroxidation. MDA inhibits ligand-binding site of serotonin receptor and therefore affects metabolism of serotonin [5, 7, 17]. Increased concentration of MDA was detected in depressed patients in comparison to healthy control. Antidepressants administration, on the other hand, decreases MDA levels [39, 47, 66].

Product of AA peroxidation is 8-iso-prostaglandine F2*α*. 8-Iso-prostaglandine F2*α* is important for stability and fluidity of cell membranes that influences consequential inflammatory response in the organism [17]. In patients with depression more than two times higher concentration of 8-iso-prostaglandine F2*α* are detected than in healthy controls. On the other hand, there does not seem to be a positive correlation between its concentration and depression severity [60, 67].

## 9. Conclusion

Major depression is associated with imbalance between several components, including neuroprogressive and neuroprotective factors (lowered concentration of BDNF [24], NF*κ*B [38], and 5-HT [5]; elevated concentration of NO [27]), antioxidants and prooxidants (increased activity of XO [35] and catalase [39], decreased activity of GPX [40] and concentration of NAC [112] and HDL [113]), proinflammatory and anti-inflammatory molecules (increased levels of proinflammatory cytokines [57], autoimmune reactions against NO-modified proteins and decreased concentration of anti-inflammatory cytokines [65]), or production of reactive species and protective mechanism of the organism. Damaging changes can be observed in structure and utility of proteins (NO-modified proteins), lipids in cell membrane (increased levels of MDA [66] and 8-iso-prostaglandine F2*α* [67]), and nucleic acids (elevated levels of 8-OxoG [32]). Formation of new epitopes and IgM mediated immune response not only indicates nitrosatively and oxidatively damaged proteins and lipids but also triggers autoimmune and inflammatory response in pathophysiology of major depression [65, 82].

Besides affecting neurotransmitters concentration in brain by antidepressants it is possible to influence or even restore impaired balance by several other substances, including dietary intake (micronutrients, vitamins, omega-3 fatty acids, and antioxidants). On the other hand, effort to artificially decrease ROS and RNS, for example, usage of NOS inhibitor as a treatment drug for major depression, should be considered with caution [74].

Further research should provide answers to several unanswered questions. Effects of antidepressants on different aspect of oxidative and nitrosative damage in patients with psychiatric disorder is not fully understood. Is oxidative stress more harmful in early or chronic state of disease? Can different markers of oxidative and nitrosative damage serve as valid* predictor* of psychiatric disorder? Can these markers distinguish between clinically different psychiatric disorders (e.g., major depression, anxiety, and schizophrenia)? Another problem is insecurity of results validation originating from blood and urine samples in comparison to values in brain or brain regions affected by disease [6]. Antioxidant effects of natural substances are also affected by discrepancies between results* in vivo *and* in vitro* [137]. Not only psychiatric disorders, but also several other diseases, for example, cardiovascular disorders, share common pathophysiological features with major depression [138]. Knowledge of connection between oxidative stress and depression is undoubtedly a step forward to better comprehension of pathophysiology of depression.

## Figures and Tables

**Table 1:** Summarizing the main findings related to oxidative stress and major depression.

Effect	Marker	Findings compared to controls	Subject	References
Neuroprogression	BDNF	↓	*Human *	[22–25]

Signaling	5-HT	↓	*Human *	[26]
	NO	↑	*Human *	[27, 28]

DNA damage	Telomerase	↓	*Human *	[29, 30]
	8-OxoG	↑	*Human *	[31, 32]

Prooxidant enzymes	XO	↑	*Human *	[33–36]
	MAO	↑	*Human *	[37]

Antioxidant enzymes	SOD	↑	*Human *	[38–40]
	GPX	↓	*Human *	[40–42]
		—	*Human *	[38, 39]
	Catalase	↑	*Human *	[39]
		↓	*Human *	[43]
	PON1	↓	*Human *	[44]

Antioxidants	CoQ10	↓	*Human *	[45]
	vit. E	↓	*Human *	[46]
	vit. C	↓	*Human *	[46–48]
	HDL	↓	*Human *	[49]

Micronutrients	Se	↓	*Human *	[50–53]
	Zn	↓	*Human *	[54, 55]

Inflammation	Proinflammatory cytokines	↑	*Human *	[41, 56–60]
	Nf*κ*B	↑	*Human *	[38]
	TRYCATs	↓	*Human *	[61–64]

Immune reaction	IgM	↑	*Human *	[65]

Lipid peroxidation	MDA	↑	*Human *	[39, 47, 66]
	8-iso-P	↑	*Human *	[60, 67]

5-Hydroxytryptamine (5-HT), 8-oxoguanine (8-oxoG), 8-iso-prostaglandin F2 alpha (8-iso-P), brain-derived neurotrophic factor (BDNF), coenzyme Q10 (CoQ10), glutathione peroxidase (GPX), high density lipoprotein (HDL), immunoglobulin (Ig), monoamine oxidase (MAO), malondialdehyde (MDA), nuclear factor *κ* B (NF*κ*B), paraoxonase 1 (PON1), superoxide dismutase (SOD), tryptophan catabolites (TRYCATs), and xanthine oxidase (XO).

## References

[1] Mathers C. D., Loncar D. (2006). Projections of global mortality and burden of disease from 2002 to 2030. *PLoS Medicine*.

[2] Caballero-Martínez F., León-Vázquez F., Payá-Pardo A., Díaz-Holgado A. (2014). Use of health care resources and loss of productivity in patients with depressive disorders seen in Primary Care: INTERDEP Study. *Actas Españolas de Psiquiatría*.

[3] Sun D., Abraham I., Slack M., Skrepnek G. H. (2014). Emergency department visits in the United States for pediatric depression: estimates of charges and hospitalization. *Academic Emergency Medicine*.

[4] Lopresti A. L., Hood S. D., Drummond P. D. (2013). A review of lifestyle factors that contribute to important pathways associated with major depression: diet, sleep and exercise. *Journal of Affective Disorders*.

[5] Leonard B., Maes M. (2012). Mechanistic explanations how cell-mediated immune activation, inflammation and oxidative and nitrosative stress pathways and their sequels and concomitants play a role in the pathophysiology of unipolar depression. *Neuroscience and Biobehavioral Reviews*.

[6] Trebatická J., Duracková Z. Psychiatric disorders and polyphenols: can they be helpful in therapy?.

[7] Scapagnini G., Davinelli S., Drago F., De Lorenzo A., Oriani G. (2012). Antioxidants as antidepressants: fact or fiction?. *CNS Drugs*.

[8] Hovatta I., Juhila J., Donner J. (2010). Oxidative stress in anxiety and comorbid disorders. *Neuroscience Research*.

[9] Gałecki P. (2014). Oxidative stress in depression. *Systems Biology of Free Radicals and Antioxidants*.

[10] Halliwell B. (2000). The antioxidant paradox. *The Lancet*.

[11] Halliwell B. (2011). Free radicals and antioxidants—quo vadis?. *Trends in Pharmacological Sciences*.

[12] Behr G. A., Moreira J. C. F., Frey B. N. (2012). Preclinical and clinical evidence of antioxidant effects of antidepressant agents: implications for the pathophysiology of major depressive disorder. *Oxidative Medicine and Cellular Longevity*.

[13] Ďuračková Z. (2014). Free radicals and antioxidants for non-experts. *Systems Biology of Free Radicals and Antioxidants*.

[14] Halliwell B. (2013). The antioxidant paradox: less paradoxical now?. *British Journal of Clinical Pharmacology*.

[15] Moylan S., Berk M., Dean O. M. (2014). Oxidative & nitrosative stress in depression: why so much stress?. *Neuroscience and Biobehavioral Reviews*.

[16] Ďuračková Z. (2010). Some current insights into oxidative stress. *Physiological Research*.

[17] Maes M., Mihaylova I., Kubera M., Uytterhoeven M., Vrydags N., Bosmans E. (2011). Lower whole blood glutathione peroxidase (GPX) activity in depression, but not in myalgic encephalomyelitis/chronic fatigue syndrome: another pathway that may be associated with coronary artery disease and neuroprogression in depression. *Neuroendocrinology Letters*.

[18] Prior P. L., Galduroz J. C. (2012). (N-3) fatty acids: molecular role and clinical uses in psychiatric disorders. *Advances in Nutrition*.

[19] Su K.-P. (2009). Biological mechanism of antidepressant effect of omega-3 fatty acids: how does fish oil act as a ‘mind-body interface’?. *NeuroSignals*.

[20] Danielsson D., Brehwens K., Halle M. (2014). Influence of genetic background and stress response on risk of mandibular osteoradionecrosis after radiotherapy of head and neck cancer. *Head & Neck*.

[21] Halliwell B., Whiteman M. (2004). Measuring reactive species and oxidative damage in vivo and in cell culture: how should you do it and what do the results mean?. *British Journal of Pharmacology*.

[22] Brunoni A. R., Lopes M., Fregni F. (2008). A systematic review and meta-analysis of clinical studies on major depression and BDNF levels: implications for the role of neuroplasticity in depression. *International Journal of Neuropsychopharmacology*.

[23] van der Meij A., Comijs H. C., Dols A., Janzing J. G. E., Voshaar R. C. O. (2014). BDNF in late-life depression: effect of SSRI usage and interaction with childhood abuse. *Psychoneuroendocrinology*.

[24] Shimizu E., Hashimoto K., Okamura N. (2003). Alterations of serum levels of brain-derived neurotrophic factor (BDNF) in depressed patients with or without antidepressants. *Biological Psychiatry*.

[25] Sen S., Duman R., Sanacora G. (2008). Serum brain-derived neurotrophic factor, depression, and antidepressant medications: meta-analyses and implications. *Biological Psychiatry*.

[26] Plein H., Berk M. (2000). Changes in the platelet intracellular calcium response to serotonin in patients with major depression treated with electroconvulsive therapy: state or trait marker status. *International Clinical Psychopharmacology*.

[27] Lee B.-H., Lee S.-W., Yoon D. (2006). Increased plasma nitric oxide metabolites in suicide attempters. *Neuropsychobiology*.

[28] Lu Y.-R., Fu X.-Y., Shi L.-G. (2014). Decreased plasma neuroactive amino acids and increased nitric oxide levels in melancholic major depressive disorder. *BMC Psychiatry*.

[29] Simon N. M., Smoller J. W., McNamara K. L. (2006). Telomere shortening and mood disorders: preliminary support for a chronic stress model of accelerated aging. *Biological Psychiatry*.

[30] Wolkowitz O. M., Mellon S. H., Epel E. S. (2011). Leukocyte telomere length in major depression: correlations with chronicity, inflammation and oxidative stress—preliminary findings. *PLoS ONE*.

[31] Wei Y.-C., Zhou F.-L., He D.-L. (2009). The level of oxidative stress and the expression of genes involved in DNA-damage signaling pathways in depressive patients with colorectal carcinoma. *Journal of Psychosomatic Research*.

[32] Maes M., Mihaylova I., Kubera M., Uytterhoeven M., Vrydags N., Bosmans E. (2009). Increased 8-hydroxy-deoxyguanosine, a marker of oxidative damage to DNA, in major depression and myalgic encephalomyelitis / chronic fatigue syndrome. *Neuroendocrinology Letters*.

[33] Herken H., Akyol O., Yilmaz H. R. (2006). Nitric oxide, adenosine deaminase, xanthine oxidase and superoxide dismutase in patients with panic disorder: alterations by antidepressant treatment. *Human Psychopharmacology*.

[34] Herken H., Gurel A., Selek S. (2007). Adenosine deaminase, nitric oxide, superoxide dismutase, and xanthine oxidase in patients with major depression: impact of antidepressant treatment. *Archives of Medical Research*.

[35] Michel T. M., Camara S., Tatschner T. (2010). Increased xanthine oxidase in the thalamus and putamen in depression. *The World Journal of Biological Psychiatry*.

[36] Kupper N., Gidron Y., Winter J., Denollet J. (2009). Association between type D personality, depression, and oxidative stress in patients with chronic heart failure. *Psychosomatic Medicine*.

[37] Sacher J., Rekkas P. V., Wilson A. A. (2015). Relationship of monoamine oxidase—a distribution volume to postpartum depression and postpartum crying. *Neuropsychopharmacology*.

[38] Lukic I., Mitic M., Djordjevic J. (2014). Lymphocyte levels of redox-sensitive transcription factors and antioxidative enzymes as indicators of pro-oxidative state in depressive patients. *Neuropsychobiology*.

[39] Gałecki P., Szemraj J., Bieńkiewicz M., Florkowski A., Gałecka E. (2009). Lipid peroxidation and antioxidant protection in patients during acute depressive episodes and in remission after fluoxetine treatment. *Pharmacological Reports*.

[40] Kodydková J., Vávrová L., Zeman M. (2009). Antioxidative enzymes and increased oxidative stress in depressive women. *Clinical Biochemistry*.

[41] Rybka J., Kędziora-Kornatowska K., Banaś-Leżańska P. (2013). Interplay between the pro-oxidant and antioxidant systems and proinflammatory cytokine levels, in relation to iron metabolism and the erythron in depression. *Free Radical Biology and Medicine*.

[42] Gawryluk J. W., Wang J.-F., Andreazza A. C., Shao L., Young L. T. (2011). Decreased levels of glutathione, the major brain antioxidant, in post-mortem prefrontal cortex from patients with psychiatric disorders. *International Journal of Neuropsychopharmacology*.

[43] Ozcan M. E., Gulec M., Ozerol E., Polat R., Akyol O. (2004). Antioxidant enzyme activities and oxidative stress in affective disorders. *International Clinical Psychopharmacology*.

[44] Bortolasci C. C., Vargas H. O., Souza-Nogueira A. (2014). Lowered plasma paraoxonase (PON)1 activity is a trait marker of major depression and PON1 Q192R gene polymorphism–smoking interactions differentially predict the odds of major depression and bipolar disorder. *Journal of Affective Disorders*.

[45] Maes M., Mihaylova I., Kubera M., Uytterhoeven M., Vrydags N., Bosmans E. (2009). Lower plasma Coenzyme Q10 in depression: a marker for treatment resistance and chronic fatigue in depression and a risk factor to cardiovascular disorder in that illness. *Neuroendocrinology Letters*.

[46] Gautam M., Agrawal M., Gautam M., Sharma P., Gautam A. S., Gautam S. (2012). Role of antioxidants in generalised anxiety disorder and depression. *Indian Journal of Psychiatry*.

[47] Khanzode S. D., Dakhale G. N., Khanzode S. S., Saoji A., Palasodkar R. (2003). Oxidative damage and major depression: the potential antioxidant action of selective serotonin-re-uptake inhibitors. *Redox Report*.

[48] Yi S., Nanri A., Matsushita Y., Kasai H., Kawai K., Mizoue T. (2012). Depressive symptoms and oxidative DNA damage in Japanese municipal employees. *Psychiatry Research*.

[49] Beydoun M. A., Beydoun H. A., Dore G. A., Fanelli-Kuczmarski M. T., Evans M. K., Zonderman A. B. (2015). Total serum cholesterol, atherogenic indices and their longitudinal association with depressive symptoms among US adults. *Translational Psychiatry*.

[50] Sher L. (2008). Depression and suicidal behavior in alcohol abusing adolescents: possible role of selenium deficiency. *Minerva Pediatrica*.

[51] Papp L. V., Holmgren A., Khanna K. K. (2010). Selenium and selenoproteins in health and disease. *Antioxidants & Redox Signaling*.

[52] Mokhber N., Namjoo M., Tara F. (2011). Effect of supplementation with selenium on postpartum depression: a randomized double-blind placebo-controlled trial. *Journal of Maternal-Fetal and Neonatal Medicine*.

[53] Conner T. S., Richardson A. C., Miller J. C. (2015). Optimal serum selenium concentrations are associated with lower depressive symptoms and negative mood among young adults. *Journal of Nutrition*.

[54] Chasapis C. T., Spiliopoulou C. A., Loutsidou A. C., Stefanidou M. E. (2012). Zinc and human health: an update. *Archives of Toxicology*.

[55] Swardfager W., Herrmann N., Mazereeuw G., Goldberger K., Harimoto T., Lanctôt K. L. (2013). Zinc in depression: a meta-analysis. *Biological Psychiatry*.

[56] Hashioka S., McGeer P. L., Monji A., Kanba S. (2009). Anti-inflammatory effects of antidepressants: possibilities for preventives against alzheimer's disease. *Central Nervous System Agents in Medicinal Chemistry*.

[57] Miller A. H., Maletic V., Raison C. L. (2009). Inflammation and its discontents: the role of cytokines in the pathophysiology of major depression. *Biological Psychiatry*.

[58] Maes M. (2002). Introduction to the special section: the depressogenic effects of cytokines: implications for the psychological and organic aetiology and treatment of depression. *The International Journal of Neuropsychopharmacology*.

[59] Song C., Halbreich U., Han C., Leonard B. E., Luo H. (2009). Imbalance between Pro- and Anti-inflammatory cytokines, and between Th1 and Th2 cytokines in depressed patients: the effect of electroacupuncture or fluoxetine treatment. *Pharmacopsychiatry*.

[60] Dimopoulos N., Piperi C., Psarra V., Lea R. W., Kalofoutis A. (2008). Increased plasma levels of 8-iso-PGF2*α* and IL-6 in an elderly population with depression. *Psychiatry Research*.

[61] Maes M. (2011). An intriguing and hitherto unexplained co-occurrence: depression and chronic fatigue syndrome are manifestations of shared inflammatory, oxidative and nitrosative (IO&NS) pathways. *Progress in Neuro-Psychopharmacology and Biological Psychiatry*.

[62] Maes M., Galecki P., Verkerk R., Rief W. (2011). Somatization, but not depression, is characterized by disorders in the tryptophan catabolite (TRYCAT) pathway, indicating increased indoleamine 2,3-dioxygenase and lowered kynurenine aminotransferase activity. *Neuroendocrinology Letters*.

[63] Catena-Dell'Osso M., Bellantuono C., Consoli G., Baroni S., Rotella F., Marazziti D. (2011). Inflammatory and neurodegenerative pathways in depression: a new avenue for antidepressant development?. *Current Medicinal Chemistry*.

[64] Ogawa S., Fujii T., Koga N. (2014). Plasma L-tryptophan concentration in major depressive disorder: new data and meta-analysis. *The Journal of Clinical Psychiatry*.

[65] Maes M., Kubera M., Mihaylova I. (2013). Increased autoimmune responses against auto-epitopes modified by oxidative and nitrosative damage in depression: implications for the pathways to chronic depression and neuroprogression. *Journal of Affective Disorders*.

[66] Sarandol A., Sarandol E., Eker S. S., Erdinc S., Vatansever E., Kirli S. (2007). Major depressive disorder is accompanied with oxidative stress: short-term antidepressant treatment does not alter oxidative—antioxidative systems. *Human Psychopharmacology*.

[67] Yager S., Forlenza M. J., Miller G. E. (2010). Depression and oxidative damage to lipids. *Psychoneuroendocrinology*.

[68] Wang L. J., Chen C. K., Hsu H. J., Wu I. W., Sun C. Y., Lee C. C. (2014). Depression, 5HTTLPR and BDNF Val66Met polymorphisms, and plasma BDNF levels in hemodialysis patients with chronic renal failure. *Journal of Neuropsychiatric Disease and Treatment*.

[69] Squassina A., Costa M., Congiu D. (2013). Insulin-like growth factor 1 (IGF-1) expression is up-regulated in lymphoblastoid cell lines of lithium responsive bipolar disorder patients. *Pharmacological Research*.

[70] Nazari M., Khodadadi H., Fathalizadeh J. (2013). Defective NF-kB transcription factor as the mediator of inflammatory responses: a study on depressed Iranian medical students. *Clinical Laboratory*.

[71] Ye N., Song Z., Zhang A. (2014). Dual ligands targeting dopamine D2 and serotonin 5-HT_1A_ receptors as new antipsychotical or anti-parkinsonian agents. *Current Medicinal Chemistry*.

[72] Dubovsky S. L., Warren C. (2009). Agomelatine, a melatonin agonist with antidepressant properties. *Expert Opinion on Investigational Drugs*.

[73] Kennedy S. H., Rizvi S. J. (2010). Agomelatine in the treatment of major depressive disorder: potential for clinical effectiveness. *CNS Drugs*.

[74] Dhir A., Kulkarni S. K. (2011). Nitric oxide and major depression. *Nitric Oxide—Biology and Chemistry*.

[75] Hickie I. B., Rogers N. L. (2011). Novel melatonin-based therapies: potential advances in the treatment of major depression. *The Lancet*.

[76] Maes M., Galecki P., Chang Y. S., Berk M. (2011). A review on the oxidative and nitrosative stress (O&NS) pathways in major depression and their possible contribution to the (neuro)degenerative processes in that illness. *Progress in Neuro-Psychopharmacology and Biological Psychiatry*.

[77] Garthwaite J. (1991). Glutamate, nitric oxide and cell-cell signalling in the nervous system. *Trends in Neurosciences*.

[78] Pinto V. L. M., Brunini T. M. C., Ferraz M. R., Okinga A., Mendes-Ribeiro A. C. (2008). Depression and cardiovascular disease: role of nitric oxide. *Cardiovascular and Hematological Agents in Medicinal Chemistry*.

[79] Maes M., Mihaylova I., Leunis J.-C. (2006). Chronic fatigue syndrome is accompanied by an IgM-related immune response directed against neopitopes formed by oxidative or nitrosative damage to lipids and proteins. *Neuroendocrinology Letters*.

[80] Kaur H., Halliwell B. (1994). Evidence for nitric oxide-mediated oxidative damage in chronic inflammation. Nitrotyrosine in serum and synovial fluid from rheumatoid patients. *FEBS Letters*.

[81] Maes M., Mihaylova I., Kubera M., Leunis J.-C. (2008). An IgM-mediated immune response directed against nitro-bovine serum albumin (nitro-BSA) in chronic fatigue syndrome (CFS) and major depression: evidence that nitrosative stress is another factor underpinning the comorbidity between major depression and CFS. *Neuroendocrinology Letters*.

[82] Maes M., Mihaylova I., Kubera M., Leunis J.-C., Geffard M. (2011). IgM-mediated autoimmune responses directed against multiple neoepitopes in depression: new pathways that underpin the inflammatory and neuroprogressive pathophysiology. *Journal of Affective Disorders*.

[83] Jun T.-Y., Pae C.-U., Chae J.-H., Bahk W.-M., Kim K.-S., Serretti A. (2003). Possible association between -G308A tumour necrosis factor-*α* gene polymorphism and major depressive disorder in the Korean population. *Psychiatric Genetics*.

[84] Antypa N., Drago A., Serretti A. (2014). Genomewide interaction and enrichment analysis on antidepressant response. *Psychological Medicine*.

[85] Maes M. (2012). Targeting cyclooxygenase-2 in depression is not a viable therapeutic approach and may even aggravate the pathophysiology underpinning depression. *Metabolic Brain Disease*.

[86] Wilkinson B. L., Landreth G. E. (2006). The microglial NADPH oxidase complex as a source of oxidative stress in Alzheimer's disease. *Journal of Neuroinflammation*.

[87] Sanmartín C., Plano D., Font M., Palop J. A. (2011). Selenium and clinical trials: new therapeutic evidence for multiple diseases. *Current Medicinal Chemistry*.

[88] de Sousa R. T., Zarate C. A., Zanetti M. V. (2014). Oxidative stress in early stage bipolar disorder and the association with response to lithium. *Journal of Psychiatric Research*.

[89] Zafir A., Banu N. (2009). Modulation of in vivo oxidative status by exogenous corticosterone and restraint stress in rats. *Stress*.

[90] Aviram M., Rosenblat M., Bisgaier C. L., Newton R. S., Primo-Parmo S. L., la Du B. N. (1998). Paraoxonase inhibits high-density lipoprotein oxidation and preserves its functions: a possible peroxidative role for paraoxonase. *The Journal of Clinical Investigation*.

[91] Kulak A., Steullet P., Cabungcal J.-H. (2013). Redox dysregulation in the pathophysiology of schizophrenia and bipolar disorder: insights from animal models. *Antioxidants & Redox Signaling*.

[92] Gomez-Pinilla F., Nguyen T. T. J. (2012). Natural mood foods: the actions of polyphenols against psychiatric and cognitive disorders. *Nutritional Neuroscience*.

[93] Ogle W. O., Speisman R. B., Ormerod B. K. (2013). Potential of treating age-related depression and cognitive decline with nutraceutical approaches: A mini-review. *Gerontology*.

[94] Rao M. N. A. (1997). Nitric oxide scavenging by curcuminoids. *Journal of Pharmacy and Pharmacology*.

[95] Hemmeter U., Annen B., Bischof R. (2001). Polysomnographic effects of adjuvant *Ginkgo biloba* therapy in patients with major depression medicated with trimipramine. *Pharmacopsychiatry*.

[96] Trebatická J., Kopasová S., Hradečná Z. (2006). Treatment of ADHD with French maritime pine bark extract, Pycnogenol. *European Child & Adolescent Psychiatry*.

[97] Chovanová Z., Muchová J., Sivoňová M. (2006). Effect of polyphenolic extract, Pycnogenol, on the level of 8-oxoguanine in children suffering from attention deficit/hyperactivity disorder. *Free Radical Research*.

[98] Dvořáková M., Ježová D., Blažíček P. (2007). Urinary catecholamines in children with attention deficit hyperactivity disorder (ADHD): modulation by a polyphenolic extract from pine bark (Pycnogenol). *Nutritional Neuroscience*.

[99] Dvořáková M., Sivoňová M., Trebatická J. (2006). The effect of polyphenolic extract from pine bark, Pycnogenol on the level of glutathione in children suffering from attention deficit hyperactivity disorder (ADHD). *Redox Report*.

[100] Bloch M. H., Hannestad J. (2012). Omega-3 fatty acids for the treatment of depression: systematic review and meta-analysis. *Molecular Psychiatry*.

[101] Sylvia L. G., Peters A. T., Deckersbach T., Nierenberg A. A. (2012). Nutrient-based therapies for bipolar disorder: a systematic review. *Psychotherapy and Psychosomatics*.

[102] Gharekhani A., Khatami M.-R., Dashti-Khavidaki S. (2014). The effect of omega-3 fatty acids on depressive symptoms and inflammatory markers in maintenance hemodialysis patients: a randomized, placebo-controlled clinical trial. *European Journal of Clinical Pharmacology*.

[103] Gertsik L., Poland R. E., Bresee C., Rapaport M. H. (2012). Omega-3 fatty acid augmentation of citalopram treatment for patients with major depressive disorder. *Journal of Clinical Psychopharmacology*.

[104] Morris M. C., Evans D. A., Tangney C. C., Bienias J. L., Wilson R. S. (2005). Fish consumption and cognitive decline with age in a large community study. *Archives of Neurology*.

[105] Parker G., Hegarty B., Granville-Smith I. (2015). Is essential fatty acid status in late pregnancy predictive of post-natal depression?. *Acta Psychiatrica Scandinavica*.

[106] Marangell L. B., Martinez J. M., Zboyan H. A., Kertz B., Kim H. F. S., Puryear L. J. (2003). A double-blind, placebo-controlled study of the omega-3 fatty acid docosahexaenoic acid in the treatment of major depression. *The American Journal of Psychiatry*.

[107] Tsai A. C., Lucas M., Okereke O. I. (2014). Suicide mortality in relation to dietary intake of n-3 and n-6 polyunsaturated fatty acids and fish: equivocal findings from 3 large US cohort studies. *American Journal of Epidemiology*.

[108] Bourre J. M. (2006). Effects of nutrients (in food) on the structure and function of the nervous system: update on dietary requirements for brain. Part 1: micronutrients. *The Journal of Nutrition, Health and Aging*.

[109] Schmelzer C., Lindner I., Rimbach G., Niklowitz P., Menke T., Döring F. (2008). Functions of coenzyme Q10 in inflammation and gene expression. *BioFactors*.

[110] Lesser G. J., Case D., Stark N. (2013). A randomized, double-blind, placebo-controlled study of oral coenzyme Q10 to relieve self-reported treatment-related fatigue in newly diagnosed patients with breast cancer. *The Journal of Supportive Oncology*.

[111] Dean O. M., Turner A., Malhi G. S. (2015). Design and rationale of a 16-week adjunctive randomized placebo-controlled trial of mitochondrial agents for the treatment of bipolar depression. *Revista Brasileira de Psiquiatria*.

[112] Berk M., Dean O. M., Cotton S. M. (2014). The efficacy of adjunctive N-acetylcysteine in major depressive disorder: a double-blind, randomized, placebo-controlled trial. *Journal of Clinical Psychiatry*.

[113] Maes M., Smith R., Christophe A. (1997). Lower serum high-density lipoprotein cholesterol (HDL-C) in major depression and in depressed men with serious suicidal attempts: relationship with immune-inflammatory markers. *Acta Psychiatrica Scandinavica*.

[114] Lehto S. M., Hintikka J., Niskanen L. (2008). Low HDL cholesterol associates with major depression in a sample with a 7-year history of depressive symptoms. *Progress in Neuro-Psychopharmacology and Biological Psychiatry*.

[115] Chen C. C., Lu F.-H., Wu J.-S., Chang C.-J. (2001). Correlation between serum lipid concentrations and psychological distress. *Psychiatry Research*.

[116] Teofilo M. M., Farias D. R., Pinto T. d. (2014). HDL-cholesterol concentrations are inversely associated with Edinburgh Postnatal Depression Scale scores during pregnancy: results from a Brazilian cohort study. *Journal of Psychiatric Research*.

[117] Äijänseppä S., Kivinen P., Helkala E.-L., Kivelä S.-L., Tuomilehto J., Nissinen A. (2002). Serum cholesterol and depressive symptoms in elderly Finnish men. *International Journal of Geriatric Psychiatry*.

[118] Barbagello M., Belvedere M., Dominguez L. J. (2009). Magnesium homeostasis and aging. *Magnesium Research*.

[119] Stewart R., Hirani V. (2012). Relationship between depressive symptoms, anemia, and iron status in older residents from a national survey population. *Psychosomatic Medicine*.

[120] Cope E. C., Levenson C. W. (2010). Role of zinc in the development and treatment of mood disorders. *Current Opinion in Clinical Nutrition and Metabolic Care*.

[121] Maes M., De Vos N., Pioli R. (2000). Lower serum vitamin E concentrations in major depression. Another marker of lowered antioxidant defenses in that illness. *Journal of Affective Disorders*.

[122] Ursini F., Bindoli A. (1987). The role of selenium peroxidases in the protection against oxidative damage of membranes. *Chemistry and Physics of Lipids*.

[123] Bottiglieri T. (1996). Folate, vitamin B12, and neuropsychiatric disorders. *Nutrition Reviews*.

[124] Nahas R., Sheikh O. (2011). Complementary and alternative medicine for the treatment of major depressive disorder. *Canadian Family Physician*.

[125] Karakuła H., Opolska A., Kowal A., Domański M., Płotka A., Perzyński J. (2009). Does diet affect our mood? the significance of folic acid and homocysteine. *Polski Merkuriusz Lekarski*.

[126] Ebesunun M. O., Eruvulobi H. U., Olagunju T., Owoeye O. A. (2012). Elevated plasma homocysteine in association with decreased vitamin B12, folate, serotonin, lipids and lipoproteins in depressed patients. *African Journal of Psychiatry*.

[127] Maes M., Fišar Z., Medina M., Scapagnini G., Nowak G., Berk M. (2012). New drug targets in depression: inflammatory, cell-mediated immune, oxidative and nitrosative stress, mitochondrial, antioxidant, and neuroprogressive pathways. And new drug candidates—Nrf2 activators and GSK-3 inhibitors. *Inflammopharmacology*.

[128] Gardner A., Johansson A., Wibom R. (2003). Alterations of mitochondrial function and correlations with personality traits in selected major depressive disorder patients. *Journal of Affective Disorders*.

[129] Berthold-Losleben M., Heitmann S., Himmerich H. (2009). Anti-inflammatory drugs in psychiatry. *Inflammation & Allergy-Drug Targets*.

[130] Salim S., Chugh G., Asghar M. (2012). Inflammation in anxiety. *Inflammation in Neuropsychiatric Disorders*.

[131] Maes M., Kubera M., Leunis J.-C. (2008). The gut-brain barrier in major depression: intestinal mucosal dysfunction with an increased translocation of LPS from gram negative enterobacteria (leaky gut) plays a role in the inflammatory pathophysiology of depression. *Neuro Endocrinology Letters*.

[132] Maes M., Kubera M., Leunis J.-C., Berk M. (2012). Increased IgA and IgM responses against gut commensals in chronic depression: further evidence for increased bacterial translocation or leaky gut. *Journal of Affective Disorders*.

[133] Maes M., Mihaylova I., Leunis J.-C. (2007). Increased serum IgM antibodies directed against phosphatidyl inositol (Pi) in chronic fatigue syndrome (CFS) and major depression: evidence that an IgM-mediated immune response against Pi is one factor underpinning the comorbidity between both CFS and depression. *Neuroendocrinology Letters*.

[134] Maes M., Leunis J. C. (2014). Attenuation of autoimmune responses to oxidative specific epitopes, but not nitroso-adducts, is associated with a better clinical outcome in Myalgic Encephalomyelitis/chronic fatigue syndrome. *Neuro Endocrinology Letters*.

[135] Maes M., de Vos N., Pioli R. (2000). Lower serum vitamin E concentrations in major depression. Another marker of lowered antioxidant defenses in that illness. *Journal of Affective Disorders*.

[136] Sugino K., Dohi K., Yamada K., Kawasaki T. (1987). The role of lipid peroxidation in endotoxin-induced hepatic damage and the protective effect of antioxidants. *Surgery*.

[137] Pun P. B. L., Gruber J., Tang S. Y. (2010). Ageing in nematodes: do antioxidants extend lifespan in *Caenorhabditis elegans*?. *Biogerontology*.

[138] Maes M., Mihaylova I., Kubera M., Uytterhoeven M., Vrydags N., Bosmans E. (2010). Increased plasma peroxides and serum oxidized low density lipoprotein antibodies in major depression: markers that further explain the higher incidence of neurodegeneration and coronary artery disease. *Journal of Affective Disorders*.

